# Specialising the parasite nucleus: Pores, lamins, chromatin, and diversity

**DOI:** 10.1371/journal.ppat.1006170

**Published:** 2017-03-02

**Authors:** Michael P. Rout, Samson O. Obado, Sergio Schenkman, Mark C. Field

**Affiliations:** 1 The Rockefeller University, New York, New York, United States of America; 2 Universidade Federal de São Paulo, São Paulo, Brazil; 3 Wellcome Centre for Anti-Infectives Research, School of Life Sciences, University of Dundee, Dundee, United Kingdom; University of Wisconsin Medical School, UNITED STATES

## Introduction

Infection by protozoan parasites remains a major cause of global human morbidity and economic hardship. With annual death rates exceeding a million people and even higher numbers afflicted by disability and compromised agricultural productivity, the organisms causing tropical diseases like leishmaniasis, trypanosomiasis, malaria, and toxoplasmosis represent an ongoing challenge. Whilst new compounds to treat malaria and toxoplasmosis have been discovered and deployed recently, this progress has not been mirrored for trypanosomiasis or leishmaniasis. Climate change, increased mobility, and mass migration also undermine our ability to control diseases caused by these organisms, and the need for new drugs to combat resistance and new strains of parasites remains acute. Nonetheless, considerable advances in understanding the cell biology of all of these infectious agents have been made, and this new knowledge is poised to contribute strongly to control strategies. In this short article, we will focus on the nuclear biology of trypanosomatid and Apicomplexan parasites, highlighting aspects that appear to represent potentially key adaptations that facilitate infection and, thus, the disease burden of these old enemies.

### Origins of the nucleus and nuclear functions

Whilst the nucleus is the defining feature of eukaryotic cells, the evolutionary origins of the organelle remain less than clear. The original architecture, composition, and, by extension, function have yet to be fully reconstructed. At the most primitive stages in eukaryotic evolution, the nucleus may well have served as a crude membranous structure enclosing the genome material (see [1]) and gathered more functionality through specialisation of the evolving nuclear envelope (NE) and the nascent nuclear contents [2]. Consisting of inner and outer NE lipid bilayers, the NE is an extension of the endoplasmic reticulum (ER); the outer membrane is contiguous with the ER, whilst the NE and ER lumenal spaces are also connected. Whilst the outer NE supports many functions in common with the ER, including, for example, the synthesis of secretory proteins, the two compartments are highly distinct both compositionally and functionally. One model implicitly assumes that the ER arose as an early feature within the nascent eukaryotic cell and subsequently diversified into the NE. Alternate models have been proposed, including a recent radical model for eukaryogenesis that suggests that the NE was originally the surface membrane of the Archaeal ancestors of eukaryotes [3–5]; thus, a full consensus model for eukaryogenesis remains to be achieved.

**Fig 1:**
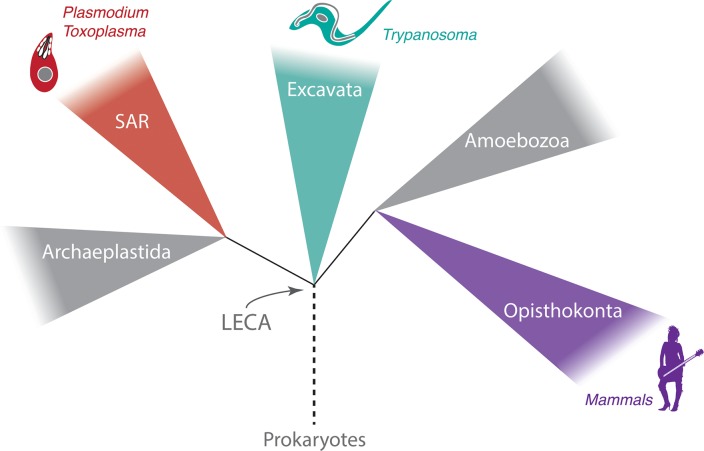
Overview of eukaryotic phylogeny emphasising the supergroup affiliation of organisms discussed here. Each of five recognised eukaryotic supergroups is shown as a coloured triangle to indicate that it contains a great many lineages, which are under continual diversification; groups not discussed are in gray, whilst Excavata (teal), stramenopiles, alveolates, and Rhizaria (SAR, red), and Opisthokonta (purple) are shown with icons for representative organisms. All of these groups radiated rapidly following the origin of eukaryotes and evolution of the LECA. Relationships are based on recent views of the branching order but should not be considered definitive.

What is clear and uncontested is that most nuclear functions associated with extant organisms, as predicted by the presence of key protein coding genes, would have been present in the last eukaryotic common ancestor (LECA) (Fig 1). Indeed, in recent years it has become apparent that far from being “primitive,” the LECA was a highly complex organism. The LECA existed well over one and a half billion years ago, providing a huge opportunity for the mechanisms that subtend basic cell functions to diversify [6]. In fact, the nucleus has a double membrane punctured by nuclear pores, nuclear pore complexes (NPCs) that fill these pores, a nucleolus responsible for ribosomal RNA transcription and ribosome assembly, heterochromatin, Cajal bodies, and other nuclear subdomains, together with a filamentous lamina subtending the NE, all of which appear to be highly conserved nuclear features. Remarkably, from a morphological standpoint, all of these features are almost invariant.

For example, by negative stain electron microscopy, the NPCs of organisms across the range of eukaryotes are extremely similar, bearing 8-fold symmetry and roughly similar dimensions. Importantly, it is not until the emergence of a fully gated NPC that the functions of the nucleus could become fully realised, as up until this point, we assumed that the NPC was able to accommodate essentially free exchange of macromolecules between the nucleoplasm and the cytoplasm [7]. Instead, modern NPCs both restrict and actively mediate the transport of different macromolecular classes [8], permitting the differentiation of the nucleoplasmic and cytoplasmic proteomes and, hence, function.

Importantly, the known protists that parasitize humans and other vertebrates are evolutionarily highly divergent from their hosts. It is therefore of great value to understand the evolutionary processes that generated this diversity. In the evolutionary history of multicellular organisms, we are very familiar with the processes of duplication, deletion, and repurposing of structures that lie at the core of the modern diversity of extant organisms. It is therefore unsurprising that identical and analogous forces, albeit at the molecular level, are at work in unicellular organisms and are important mechanisms underpinning the diversification of protozoa.

**Fig 2:**
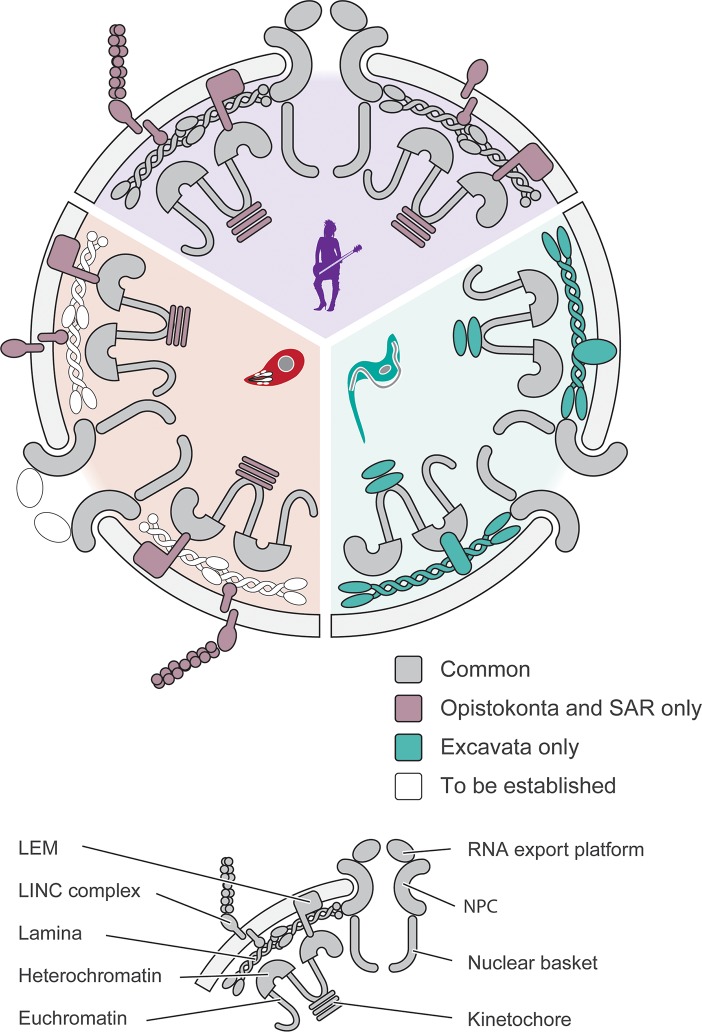
Conservation and divergence at the nuclear envelope. The major protein and nucleic acid complexes responsible for control of gene expression, nucleocytoplasmic transport, and regulation of nuclear architecture are shown. The circular nucleus diagram is divided into three colourised sectors that correspond to those of Fig 1. Elements are colourised that are known to deviate from likely LECA components, whilst unknown elements are shown as open symbols. Mixed purple/green is used to designate factors that are shared between Opisthokonts and Apicomplexa. Significantly, the extensively studied *Homo sapiens* nucleus appears to retain much of the machinery of the LECA, whilst trypanosomes have several clear examples of divergent molecular systems that subtend nuclear functions. In Apicomplexa, the basic nuclear system appears once more to be similar to the LECA, although several aspects (for example, the composition of the nuclear pore complex and the identity of the lamina) remain unknown at this time; evidence suggests that Apicomplexa do not possess a LECA/mammalian type lamina, suggesting the presence of a novel machinery awaiting discovery.

Two lineages account for the major proportion of species of parasitic protozoa: the Apicomplexa (*Toxoplasma gondii* and *Plasmodium* spp.) residing within the SAR supergroup and the Kinetoplastida (*Trypanosoma* and *Leishmania*) located within the Excavata supergroup. Additional highly important parasites, including *Naegleria*, *Giardia*, and *Trichomonas* are also Excavates [9] (Fig 1). Each of these supergroups diversified rapidly following the emergence of the LECA, and notwithstanding the high degree of morphological conservation of the nucleus, even by these protists, the molecular mechanisms that underpin nuclear functions appear to be divergent, albeit frequently subtending similar processes (Fig 2). Hence, it is essential to understand the molecules involved in these various functions, as the simple observation of cellular activities and structures can provide a false impression of a high degree of conservation, when in fact, the molecular mechanisms subtending them are distinct.

### Holding it together—The lamina

In metazoa, the structural organisation of the nucleus is supported by a filamentous protein network at the inner face of the NE. Principal components of this system are ~60 kDa coiled-coil lamin proteins [10]. Lamin expression in differentiated cells is required to support nuclear architecture, prevent abnormal blebbing of the NE [10, 11], and position the NPCs [12, 13]. Originally believed to be metazoan-restricted and, hence, a lineage-specific mechanism for multiple nuclear activities [10], lamin orthologs are actually present across a wide range of eukaryotes and most likely represent the configuration in the LECA [14, 15] (Fig 2). It is, however, clear that the lamin system cannot be universal, as (for example) *Saccharomyces cerevisiae* lacks lamins [16], almost certainly due to a lineage-specific loss; instead, several distinct proteins, including Mlp1 and Esc1, appear to have partially subsumed the functions of the lamina [17–20].

Both *Plasmodium* and African trypanosomes exploit heterochromatin, much of it associated with the nuclear periphery, to control gene expression. Specifically, both organisms possess a system of antigenic variation that relies on achieving switchable monoallelic expression: *var* gene products in *Plasmodium* and variant surface glycoproteins (VSGs) in trypanosomes.

In trypanosomes, two large coiled-coil proteins, NUP-1 (450 kDa) and NUP-2 (170 kDa), are major components of the nuclear lamina that are involved in maintaining silent VSG genes at subtelomeric expression sites in a state of very low transcriptional activity [21, 22]. Additionally, both also participate in repression of procyclin, the major antigen expressed in the insect stage, in the mammalian-infective form; significantly, both VSGs and procyclin are transcribed by RNA polymerase I, which sets both of these loci apart from the bulk of protein coding genes, which are transcribed by RNA Pol II. It remains to be understood how the mechanisms of transcription, chromatin modification, and silencing connects with this lamina at the molecular level, but at the cellular level, the role in maintaining a structure that allows segregation of chromatin into peripheral heterochromatin is likely critical. Further, as NUP-1 and NUP-2 are conserved across trypanosomes, this suggests a similar system is present in many pathogenic protozoa [14, 22]. In every important structural and functional sense examined, NUP-1 and NUP-2 both behave similarly to lamins. The extreme divergence in size and sequence between NUP-1/NUP-2 and lamins, considered alongside their similar coiled-coil architecture and other structural features, has made it impossible to determine if these two systems arose via an extreme case of evolutionary divergence or are an example of convergence (Fig 2).

Heterochromatin-based silencing in trypanosomes involves several proteins, many of which are well conserved with the opisthokont host, such as SIR2, ISWI, RAP1, and histone deacetylase (DAC) 3 [23–25]. In the insect-infective stage, trypanosome telomeres tend to be close to the nuclear periphery [21], but this is much less pronounced in the mammalian-infective forms. Basal telomeric silencing also invokes a second deacetylase, DAC1, while histone H1 participates in maintaining condensed chromatin in silenced regions [26–28]. The single active VSG gene is transcribed exclusively at the expression site body (ESB), a specific nuclear subdomain that avoids the nuclear periphery and likely removes the active VSG from these regions of chromatin modification and repression [29]. Significantly, *Trypanosoma brucei* lacks H3K9me3, which is a well-documented marker for heterochromatin. Further, while TbSIR2 is involved in the silencing of genes adjacent to telomeres, it remains to be demonstrated that this is required for monoallelic expression of VSG and, hence, antigenic variation [30], although, given the evidence, it is perhaps likely.

Whilst some species within the Alveolata do possess lamin orthologs, along with several lamin-binding proteins [14], this does not include the Apicomplexa, and at present, no Apicomplexan lamina component has been identified. In *Plasmodium*, a single *var* variant is expressed from a subtelomeric site and, similarly to trypanosomes, this also involves specific histone modifications [31, 32]. The limited information available suggests that *Plasmodium* retains a chromatin structure that is more similar to the Opistokhonta than the trypanosomes. For example, PfSIR2 has been implicated in *var* silencing, and *Plasmodium* retains the H3K9me3 histone modification, which is also involved in silencing *var* [32, 33]. H3K9me3 is associated with transcriptionally silent genes, including most *var* gene loci. Again, similarly to trypanosomes, PfSIR2 and a PfDAC have been implicated in control of this process, together with a conserved heterochromatin protein (HP) 1 and SET (Su(var)3-9, Enhancer-of-zeste, and Trithorax) domain protein [31, 34–36]. What clearly differentiates the *Plamodium var* mechanism from the trypanosome VSG mechanism is that the active *var* gene remains in a nuclear peripheral location, rather than being relocated to a specialised structure within the nuclear interior. It is also the case that the *var* expression site is not a limiting factor for mutually exclusive expression and can accommodate more than one active *var* promoter at a time, unlike African trypanosomes.

Differently from Trypanosomes, *Plasmodium* display a typical heterochromatin protein (HP1) that interacts with the heterochromatin histone mark H3K9me3 [37] and associates with both subtelomeric regions, as well as additional loci that are strongly developmentally regulated. Telomeres in *Plasmodium* are clustered, which also appears to be different from trypanosomes, although the presence of hundreds of minichromosomes has made understanding telomere dynamics for conventional chromosomes especially difficult in trypanosomes. The number of puncta visualised with telomere–repeat probes in trypanosomes is substantially less than the ~250 telomeres present in the trypanosome nucleus, suggesting some telomeric clustering is at play, but the precise level of organisation of these chromosomal subdomains remains to be fully elucidated. Regardless, the active plasmodial *var* is separate from the remaining clustered telomeres and suggests that the nuclear periphery is able to accommodate both active and inactive chromatin, which is also the case in higher eukaryotes [38]. However, this appears to have nothing to do with NPC-mediated activation of chromatin, as NPCs and *var* expression sites appear distinct [39].

Both of these examples are of significant interest for at least three reasons. First, the high level of divergence from the host lamina system (on the one hand, an identified cohort of proteins [in the case of trypanosomes], and on the other, a yet to be determined set of components for *Plasmodium*) may provide druggable components, as their parasite-specific nature could provide significant specificity. Second, it is clear that these parasites are exploiting highly conserved mechanisms for the definition of heterochromatin, which also likely points to ancient origins at the core of these processes. Third, the organisation and positioning of nuclear components, including the NPC and heterochromatin, are extremely similar between the parasite and host in terms of overall function but are clearly mediated by distinct molecular mechanisms (Fig 2). Indeed, in trypanosomes, with the exception of the NPC, most of the otherwise conserved proteins associated with the NE appear absent [14]. Significantly, how the LECA lamina (based on lamins) came to be a system supported by NUP-1/NUP-2 and the identity of the currently cryptic lamina system in Apicomplexa remain to be determined.

### Getting in and out: The nuclear pore complex

The nuclear envelope is fenestrated by nuclear pores, in which are assembled NPCs that facilitate the bidirectional exchange of proteins and nucleic acids between the nucleoplasm and cytoplasm. NPCs consist of about 30 distinct proteins, but the presence of multiple copies means the total number of polypeptides present is over 500 in yeasts [40] and likely even greater in metazoa. High-resolution reconstructions, based on a combination of X-ray crystallography, analysis of protein–protein interactions, and subunit geometry, together with immuno- and cryo-electron microscopy have provided increasingly sophisticated views of the NPC’s structure [41–43]. At its simplest, the NPC possesses a central channel filled with intrinsically disordered and highly mobile phenylalanine-glycine (FG)-containing proteins. The channel is constructed of subcomplexes arranged in rings that form inner and outer scaffolds and that serve to bend and stabilise the nuclear pore membrane as well as act as anchors for the FG proteins. Finally, at the cytoplasmic and nuclear faces of the NPC are fibrous structures referred to as cytoplasmic fibrils and the nuclear basket, respectively. Both are important in the transport, processing, and quality control of RNAs, which are translocated in a complex with a large cohort of proteins [44].

Much of the NPC scaffold is comprised of β/α-fold secondary structural proteins, which bear a clear resemblance to proteins of the vesicular transport and the intraflagellar transport systems [45–47]. This has been proposed as an evolutionary link between the NPC and these other processes and one that may explain many aspects of eukaryogenesis [1, 46]. Significantly, all of these systems are present in the LECA, which therefore indicates that differentiation of the NPC was an early event in the evolution of the eukaryotic cell. Furthermore, the largest transport receptor family, the karyopherins, which are responsible for recognition of nuclear localisation and nuclear export signals and translocation across the NPC, also appear to have been rather well conserved and are also related to some NPC inner scaffold nucleoporins, as well as vesicular transport proteins [48, 49], and, for the most part, were well established by the time of the LECA [1].

Until recently, the full protein composition and subunit arrangement of NPCs of only two organisms were known: yeast and vertebrates [40, 50–52], essentially close cousins within eukaryotic diversity. Comprehensive lists of proteins comprising the higher plant NPC [53] and trypanosome NPC [54] were also described, but complete composition and subunit arrangements were lacking. Both of these datasets indicated that NPCs are well conserved across eukaryotes and that, despite considerable sequence diversity, the proteins present bore remarkably conserved β/α-fold secondary structures.

Although the absence of complete data has precluded detailed reconstructions, we recently described the full protein composition and overall protein–protein interaction map for the *T*. *brucei* NPC [55]. These new data began to unravel some of the evolutionary events and specialisations that reside within the NPC (Fig 2). Considered together with comparisons between yeast and human NPCs, as well as additional taxa, it is now clear that while the proteins and complexes making up the NPC are quite conserved, their arrangements can differ greatly between different cells in the same organism or even different nuclei in a single cell [51, 56, 57]. Whether there are NPC compositional or other structural changes that accompany differentiation or development in parasites is currently unknown but certainly of interest and direct relevance to understanding the modulation of gene expression.

The number and positioning of NPCs does not appear to vary significantly between the two major life stages of *T*. *brucei* (i.e., the insect and bloodstream form), likely reflecting that both are highly proliferative and therefore have very active transcription. Whilst the overall number of nucleoporins present within the trypanosome NPC is similar to that in animals and fungi, it does lack several subunits [55]. These losses are almost exclusively at the cytoplasmic face. Significantly, many components required for the ATP-driven export of mRNA, which includes the RNA export factor Gle1 and ATP-dependent DEAD box helicase Dbp5, together with their NPC docking sites, are absent [55]. This, then, indicates a distinct export mechanism and raises the question of how mRNA export operates in trypanosomes. Furthermore, the FG-repeat proteins are configured rather differently. Not only are the positions of the repeat regions distinct from those in animals and fungi, but the proteins are arranged in a symmetric manner with respect to the NPC and the nuclear/cytoplasmic axis, in contrast to higher eukaryotes, where there is evidence for bias in FG-repeat protein localisation [7, 55].

Whilst the precise functional consequences of these alterations are presently unknown, we propose that the absence of the Gle1/Dbp5 system is most likely connected to mRNA export and the rather distinct mechanism of transcription in trypanosomes. In trypanosomatids, most transcripts are produced as part of polycistronic transcription units, and mature mRNAs are produced by cleavage and *trans*-splicing. Furthermore, with the exception of two genes, trypanosome genes are intron-free [58, 59]. Importantly, this has the consequence that essentially no *cis*-splicing takes place, and hence, mRNA processing is potentially less complex than in higher eukaryotes, as the need to quality control and to resolve alternate splicing or lariat splicing in intermediate structures is absent. However, at present, this proposal is tentative and will require characterisation of the NPCs from related organisms such as *Euglena*, in which mRNAs are processed by both *cis-* and *trans*-splicing.

Several additional aspects of NPC function also appear to be present in trypanosomes, including an association of nuclear basket components with the mitotic spindle and the presence of FG-repeat proteins at regions of high transcriptional activity within the nucleoplasm and where they may participate in mRNA processing [60, 61]. Overall, this indicates that, as with higher eukaryotes [62], the NPC of trypanosomes is deeply embedded within many nuclear functions, having influences on many aspects of gene expression in addition to its central function in nucleocytoplasmic transport.

Substantially less is known concerning the NPC composition of *Plasmodium* and *Toxoplasma* beyond the identification of a small number of conserved NPC proteins [63, 64]. In addition, there is intriguing evidence for the evolution of novel Nups in *Plasmodium* by gene fusion. Specifically, Sec13 in several *Plasmodium* species is substantially larger than most other organisms (~90 kDa versus ~40 kDa, respectively) and appears to be the result of additional coding sequence homologous to Nup145 present in a separate intron at the C-terminus of the Sec13 β-propeller [63]. Intriguingly, this is quite variable between different species of *Plasmodium*, which may indicate a level of ongoing selection (and hence, adaptation) across the lineage.

A correlation between transcription and NPC number is well known in metazoa, and the number of NPCs varies between life stages during the intraerythrocytic position of the life cycle in *Plasmodium falciparum*, which is likely also connected to transcriptional activity [65]. Interestingly, NPC number also correlates with nuclear volume and is highest in the trophozoite and early schizont stages and lowest at late schizont stages of the erythrocyte infection cycle. In early ring forms, each *P*. *falciparum* nucleus bears very few NPCs, and these are clustered at one pole of the nucleus, suggesting a lamina or other organisational system must be present within *Plasmodium* nuclei. Significantly, NPC number also correlates with the presence of histone modifications associated with more open chromatin and, hence, transcriptional activity. PfSec13 localisation suggests that the NPCs of intraerythrocytic stages do not associate with heterochromatin, as they do not colocalise with HP-1 or H3K9me3 [63]. During the latter stages of schizogony, the number of NPCs per nucleus decreases, which may simply be a dilution of existing NPCs between daughter cells, indicating that ongoing NPC synthesis has ceased [65].

### Moving chromosomes around: The kinetochore

Another example of distinct molecular mechanisms operating in parasite nuclei is the unconventional cohort of proteins comprising the trypanosome kinetochore, which lack canonical centromeric proteins such as the centromere-specific variant histone H3 (CenH3 or CENP-A), considered the epigenetic marker of centromeres in higher eukaryotes [66]. Remarkably, although the trypanosome kinetochore is unconventional, it still mediates chromosome segregation by interacting with centromeric regions and mitotic spindle microtubules [67]. Centromeres in trypanosomes have been mapped [67–69] and, in *T*. *brucei*, are composed of adenine/thymidine-rich 147 bp repeats that stretch across regions of 20–120 kb and are associated with transposable elements [68, 70]. This is similar to mammalian centromeres, which also consist of AT-rich α-satellite repeats disrupted by retrotransposons and stretch over several megabases [71]. By contrast, centromeres in the American trypanosome *T*. *cruzi* are centered on guanosine/cytosine-rich regions of ~10–20 kb that are comprised of degenerate retroelements [69]. Centromeres in the related kinetoplasid *Leishmania* remain uncharacterised [72–74].

*P*. *falciparum* centromeres have been mapped to 2 kb repeat regions that are extremely AT-rich (98%) and are identical in size and sequence on all chromosomes [75, 76]. There, the similarity to trypanosomes ends, as *P*. *falciparum* has orthologs of canonical centromere-specific proteins, such as CenH3 and the DNA-binding CENP-C protein, that constitutively associate with centromeres in higher eukaryotes [75, 77–81]. As expected, PfCenH3 and PfCENP-C, in conjunction with histone H2AZ, localise to *Plasmodium* centromeres [82]. Furthermore, *P*. *falciparum* CenH3 can complement the yeast CenH3 ortholog, Cse4p [83]. The related organism *T*. *gondii* also possesses CenH3, suggesting that conventional kinetochores are a conserved feature in apicomplexans [84] (Fig 2). TgCenH3 associates with centromeres, which cluster together at the centrocone, a unique, specialised spindle pole body that constitutively associates with the nuclear envelope throughout the cell cycle [84–86].

### Nuclear positioning

Connections between the nucleus, the lamina, and the cytoskeleton are essential for positioning the nucleus [87, 88]. In mammals, these involve the LINC (Linkers of Nucleoskeleton and Cytoskeleton) complex, which bridges both outer and inner nuclear membranes and connects the lamina with the cytoskeleton; the LINC complex is comprised of a SUN (Sad1p, UNC-84) domain protein on the inner NE and a KASH (Klarsicht, ANC-1, Syne Homology) domain protein on the outer NE [89–91], while SUN domain proteins provide a physical link to lamins and nuclear pore complexes [92, 93]. Though SUN domain proteins are widely distributed and predicted in all eukaryotic supergroups, the single SUN domain protein in trypanosomes is distinct from the NE-associated subfamily [94]. KASH domain proteins are widely distributed but, again, are absent from trypanosomes (Fig 2). Involvement of the actin and tubulin cytoskeleton with the LINC complex is very clear in metazoa, as is participation of several KASH domain NE proteins, e.g., Nesprin1 and 2G and Anc-1 [89, 95, 96].

During their life cycles, many parasitic protozoa undergo several major morphological changes, and trypanosomes and Apicomplexa are no exception. The relative positioning of the nucleus in the trypanosome cell is highly precise and indeed has been used classically to define specific life stages [97, 98]. This is likely associated with overall mechanisms of organelle segregation in trypanosomes, which are extremely ordered. This appears to be an adaptive mechanism that may be important for meeting the need to accommodate large numbers of cells within a host cell, as in the case of *T*. *cruzi* amastigotes, for example. Morphological changes could also arise as a consequence of the type of movement required for adaptation to the environment. It is significant that the nucleus of *T*. *cruzi* trypomastigotes, a nonproliferative but infective stage, becomes elongated and enriched in heterochromatin-like structures without a defined nucleolus [99, 100]. Because *T*. *cruzi* trypomastigotes attach and actively invade mammalian cells but do not divide, these cells mainly restrict synthetic activity to maintaining surface components that interface with the host cell [101]. These changes are probably consequences of a low state of transcription [99] and the presence of unique post-transcriptional modification of histones and proteins [102]. When *T*. *cruzi* infective forms regain a nutrient-rich environment, a set of signaling events occur, and the nucleus returns to its original spherical shape. With the absence of a trypanosome LINC complex, how these positioning and structural changes are achieved has no obvious molecular basis. One gene product in *T*. *brucei*, TbAIR9, does affect nuclear positioning and localises to the subpellicular array, but additional impact on the overall cell dimensions makes its precise role unclear [103]. Similarly, in Apicomplexa, where there are LINC complexes but no known lamina, no factors affecting nuclear position are known. In *T*. *gondii* tachizoites, the position of the nucleus is also rather stable, normally being positioned in the third of the cell distal to the conoid. Significantly, the NE also bears the major ER exit sites [104], and arrangement of organelles is quite precise, but the molecular mechanisms that govern nuclear positioning remain unknown.

### Perspectives

The emergence of the nucleus is a pivotal event in evolution and occurred over one and a half billion years ago. Given such a huge gulf of time between this origin and the present day, there have been ample opportunities for the acquisition of new and diverse nuclear roles by different eukaryotic lineages. Parasitic protists, which have experienced considerable adaptive pressures and frequent bottlenecking during transmission (which can increase the rate of fixation of specific alleles) represent potentially excellent windows into such diversity. How the nucleus, this ancient aspect of the eukaryotic cell, has changed over such immense stretches of time can inform the manner in which these lineages have produced pathogenic or adaptive mechanisms linked to their parasitic needs. What has emerged recently, by considering the nuclear pore complex, the nuclear lamina, and several additional aspects of nuclear biology, is a melange of change and stasis that nevertheless may also reflect significant evolutionary and functional rigidity, restricting how diverse nuclear structures can become.

Within the trypanosome NPC, we have uncovered considerable diversity (in particular, aspects potentially integrated within RNA export systems, as well as possibly transcriptional control and genome segregation). A recurrent theme is the apparent subtending of similar functions by diverse proteins, although the precise events behind these novel mechanisms remain to be uncovered. In the case of the lamina, where evidence indicates a novel system in trypanosomes and likely also in Apicomplexa (as evidenced by the absence of any of the known lamina systems), the systems of heterochromatinisation, NPC positioning, chromosome segregation, and telomeric positioning all appear retained, yet in some cases, they are mediated by distinct groups of proteins. Despite this, it appears that the Aplicomplexa retain a more canonical system overall. This may reflect their greater reliance on promoter-based gene expression, as opposed to polycistronic mechanisms. The polycistronic mode of transcription can also have a profound impact on genome organisation (for example, the retention of genes and gene order within polycistronic transcription units between different kinetoplastids, despite overall reorganisation of the genome).

Furthermore, trypanosomatids have evolved a solution to the accurate segregation of a very large number of chromosomes, together with a simpler program of *trans*-splicing for mRNA maturation and the nonconventional use of RNA Pol I for transcription of high-abundance surface antigens, which includes VSGs. Both of these latter aspects may be connected with a need for rapidity in mRNA processing, and it is possible that simple alternate *trans*-splicing is important for the rapid switch in gene expression required to adapt to a new host. Furthermore, African trypanosomes rely extensively on the need for monoallelic expression of VSGs, but such strict control of *var* gene expression does not seem to be the case for *Plasmodium*. Whilst it remains unclear how precisely to exploit these novel biological aspects for therapeutics, if suitable protein–protein interactions or enzymatic activities can be identified, these processes may well represent attractive targets for drug development. Finally, understanding how these diversifications contribute to pathogenesis and the success of parasitic protists remains a challenge for the future.
